# Case report: Suspicious laryngeal mass: a case of laryngeal lymphoma misdiagnosed as chronic inflammation

**DOI:** 10.3389/fonc.2024.1424785

**Published:** 2024-07-29

**Authors:** Juanjuan Hu, Baoai Han, Ranran Ding, Yue Qiu, Haiying Sun, Yun Zhu

**Affiliations:** ^1^ Department of Otorhinolaryngology, Union Hospital, Tongji Medical College, Huazhong University of Science and Technology, Wuhan, China; ^2^ Department of Otorhinolaryngology, Head and Neck Surgery, Zhongnan Hospital of Wuhan University, Wuhan, China; ^3^ Department of Pathology, Union Hospital, Tongji Medical College, Huazhong University of Science and Technology, Wuhan, China

**Keywords:** laryngeal lymphoma, tongue base, chronic tonsillitis, cervical lymphadenopathy, differential diagnosis

## Abstract

This case report aims to highlight the importance of considering lymphoma as a potential differential diagnosis in patients presenting with laryngeal mass and associated cervical lymphadenopathy, particularly those with a history of chronic tonsillitis. A case of a 63-year-old male patient who underwent bilateral tonsillectomy for tumor in the left tonsil was presented. Two months after the procedure, he developed throat discomfort, dysphagia, neck swelling, and other symptoms. The patient was initially diagnosed with “tongue base mass” and chronic lymphadenitis. Partial excision of the tongue base mass was performed twice in another hospital, revealing chronic inflammation of the epithelial mucosa. Further evaluations, including electron laryngoscopy and imaging studies, were conducted to investigate the condition. A computed tomography (CT) scan showed irregular soft tissue density in the oropharyngeal region, along with multiple lymph nodes in the neck. Subsequent histopathological examination of the lingual base biopsy revealed peripheral T-cell lymphoma with a follicular T-helper cell phenotype. Immunohistochemical staining confirmed specific markers while ruling out other markers. *In situ* hybridization testing demonstrated positivity for Epstein–Barr virus-encoded RNA, and TCRG clonality was confirmed. The duration from symptom onset to diagnosis was 2 months. This case emphasizes the importance of considering lymphoma in patients with laryngeal mass and associated cervical lymphadenopathy, especially when a history of chronic tonsillitis is present. Accurate diagnosis and early intervention are crucial for effective management and improved patient outcomes.

## Introduction

Primary lymphoma of the larynx is extremely rare, accounting for approximately 1% of all laryngeal tumors (1–3). Laryngeal lymphoma represents a distinct entity among laryngeal malignancies. It is more commonly seen in men and typically affects individuals in their sixth to seventh decade of life (4). It consists mainly of non-Hodgkin lymphomas (NHLs). Extranodal natural killer/T-cell lymphoma, located in the larynx, is a rare condition that accounts for <11% of all lymphomas (5).

The clinical presentation of laryngeal lymphoma can vary, making its diagnosis challenging. Patients may present with symptoms such as hoarseness, dysphagia, or airway obstruction (3). The initial presentation can often mimic benign conditions like inflammation or infection, leading to misdiagnosis and delays in appropriate management (5). Therefore, it is crucial for clinicians to maintain a high index of suspicion for laryngeal lymphoma when evaluating patients with persistent or atypical laryngeal symptoms.

Here, we present a 63-year-old man with T-cell lymphoma of the larynx that was misdiagnosed with inflammation even after several biopsies from the primary laryngeal lesion. The final diagnosis of T-cell lymphoma was obtained in our hospital 2 months after the first biopsy from the primary site. Here, to emphasize the importance of considering laryngeal lymphoma in the differential diagnosis of laryngeal tumors, we report a rare case of primary laryngeal lymphoma and discuss its characteristics and difficulty of diagnosis.

## Case presentation

### Clinical history

The Ethics Committee of Tongji Medical College, Huazhong University of Science and Technology approved this study (IORG No. IORG0003571). This case report was prepared in accordance with the CARE (Case Reports) guidelines, as available on the EQUATOR Network (https://www.equator-network.org/). A 63-year-old male patient presented to the Ear, Nose, and Throat (ENT)Department with complaints of a foreign body sensation in the throat and dysphagia. The symptoms emerged 2 months after undergoing bilateral tonsillectomy for chronic tonsillitis. The patient also reported swelling in the right side of the neck, along with voice changes. There was an absence of throat pain or respiratory distress. The patient had a fever but did not experience weight loss or night sweats. Before the tonsillectomy, his tonsils were symmetrically enlarged to grade II, and postoperative pathology indicated chronic tonsillitis. The patient visited the Guizhou Provincial Staff Hospital, where he initially received a diagnosis of “tongue base mass” and chronic lymphadenitis. He visited our hospital for evaluation and treatment in March 2023.

### Flexible laryngoscopy

**Figure 1:**
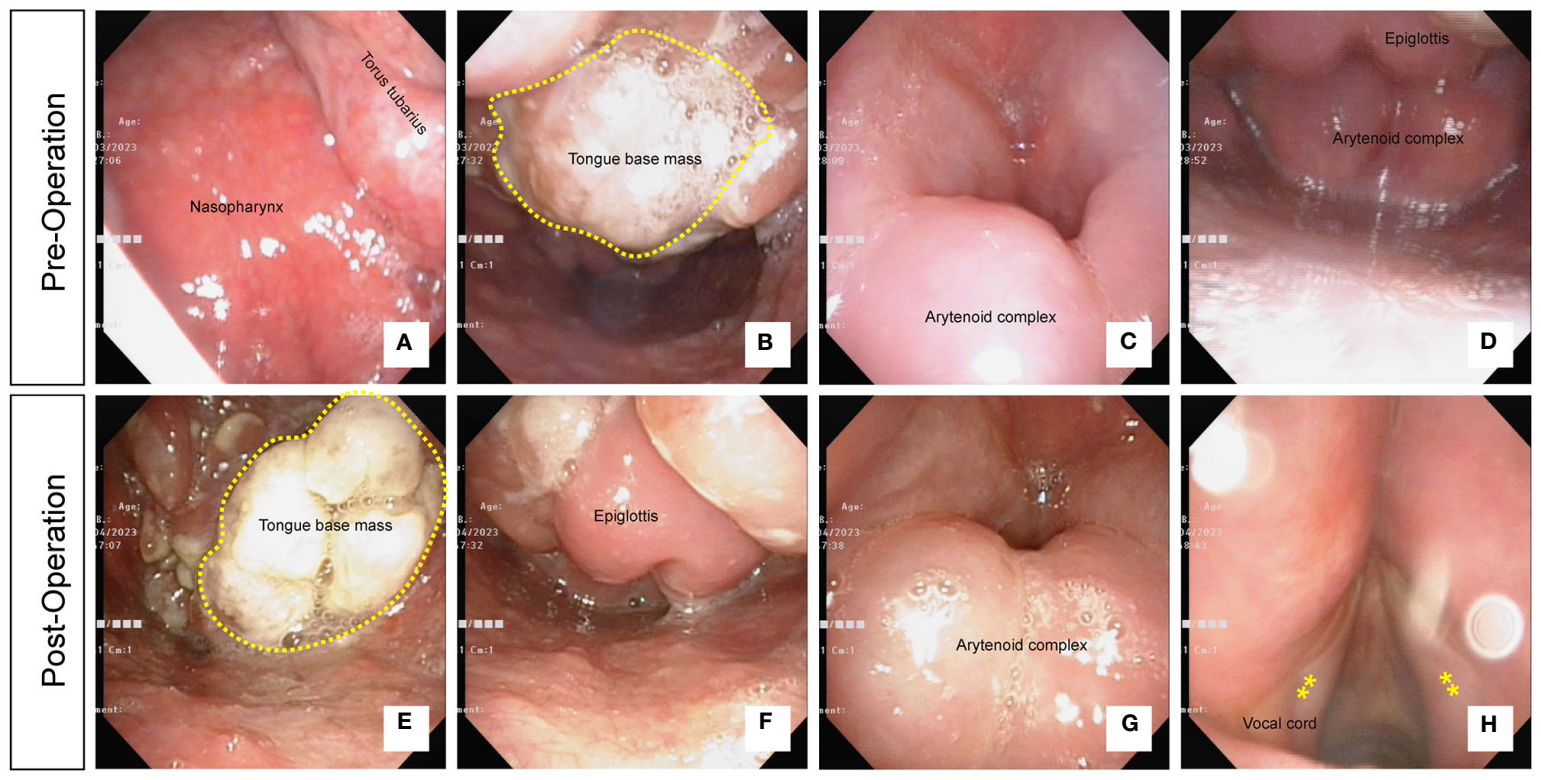
**(A)** Flexible laryngoscopy showing slight hypertrophy of the nasopharynx with symmetrical fossa of rosenmuller. **(B)** A large mass in the base of the tongue, predominantly on the right side. **(C)** Obvious edema observed in the submucosa of the epiglottis. **(D)** Edema in the arytenoid complex obstructing the respiratory passage. **(E)** A reduction in the size of the mass in the base of the tongue following 2 weeks of biopsy. **(F)** Persistent swelling observed in the epiglottis. **(G)** Persistent swelling observed in the arytenoid complex. **(H)** Smooth surface and normal morphology of the vocal cords.

Flexible laryngoscopy demonstrated a slight hypertrophy of the nasopharynx, with symmetrical fossa of rosenmuller (**Figure 1A**). Additionally, there was a large mass in the base of the tongue, predominantly on the right side (**Figure 1B**). Furthermore, there was obvious edema in the submucosa of the epiglottis and arytenoid complex, leading to obstruction of the respiratory passage (**Figures 1C, D**).

After the biopsy of the mass in the tongue base, we initiated 1 week of hormone therapy and 2 weeks of antibiotic treatment to reduce inflammation. After this course of treatment, a follow-up examination using flexible laryngoscopy was performed. The results showed that compared to the pretreatment findings, the mass in the base of the tongue had decreased in size (**Figure 1E**). However, there was persistent swelling in the epiglottis and arytenoid complex (**Figures 1F, G**). The surface of the vocal cords appeared smooth and exhibited a normal morphology (**Figure 1H**).

### Imaging

**Figure 2:**
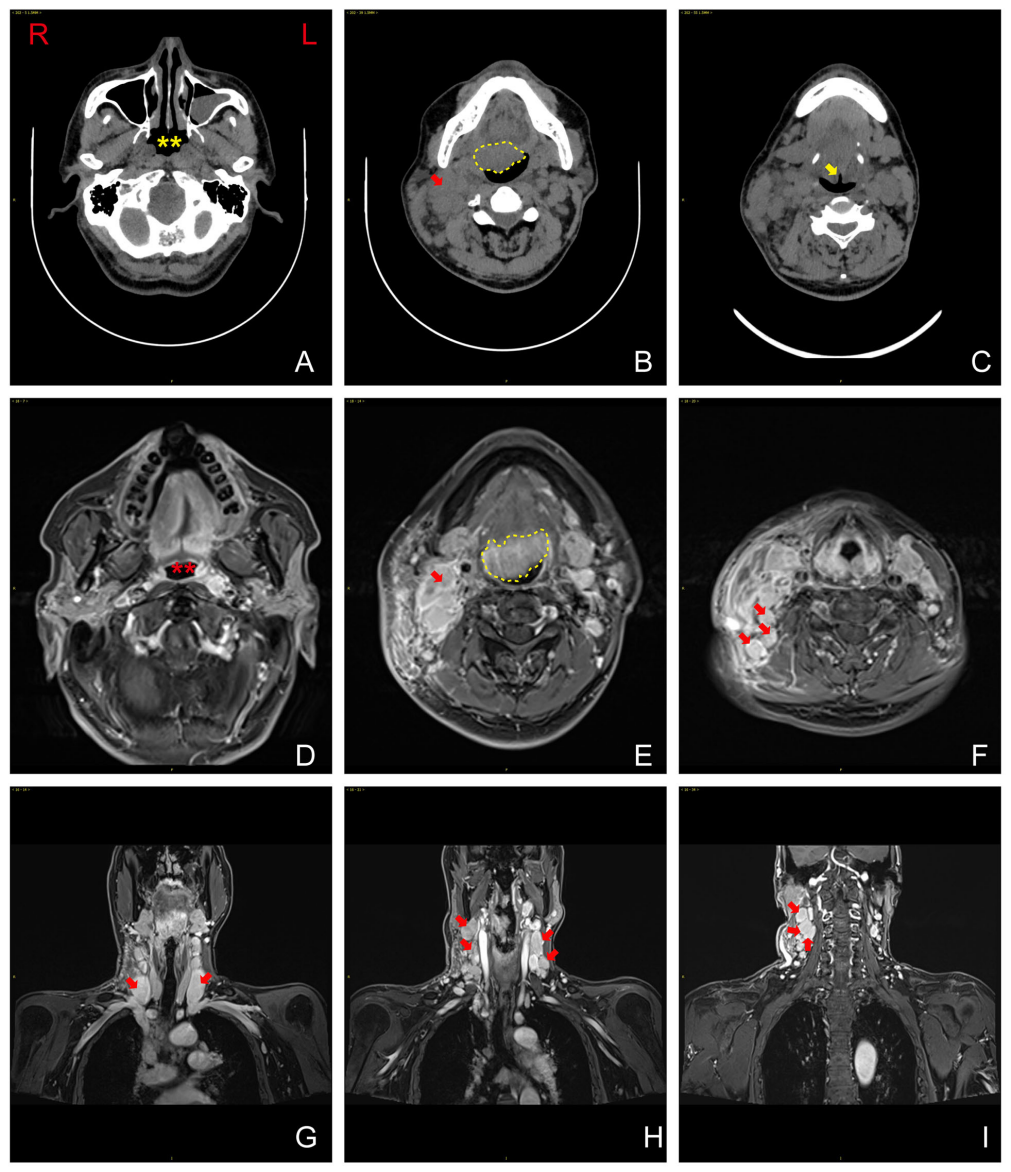
**(A)** Computed tomography (CT) imaging revealing nearly normal nasopharynx. Yellow asterisks show the nasopharynx. **(B)** Irregular, poorly defined boundary soft tissue observed in the oropharyngeal region (yellow dash line). **(C)** Obvious swelling of the epiglottis (yellow arrowhead). **(D)** Magnetic resonance imaging (MRI) revealing no significant soft-tissue thickening in the bilateral walls of the oropharynx (red asterisks show the oropharynx). **(E)** A mass in the base of the tongue (yellow dash line). **(F–I)** Uniform enhancement in bilateral neck lymph nodes and larger lymph nodes (red arrowhead). R, right; L, left.

A preoperative computed tomography (CT) imaging showed an almost normal nasopharynx (**Figure 2A**). There was an irregular, poorly defined boundary soft tissue in the oropharyngeal region (**Figure 2B**). In addition, we also observed an obvious swelling of the epiglottis (**Figure 2C**).

A magnetic resonance imaging (MRI) was performed after the biopsy. No significant soft-tissue thickening was observed in the bilateral walls of the oropharynx (**Figure 2D**). Furthermore, the imaging showed that in the base of the tongue to the epiglottis region, there was a mass, measuring approximately 36 mm × 31 mm (**Figure 2E**). The lesion extended downward, involving the bilateral aryepiglottic folds. Significant enhancement was observed in the corresponding area on contrast-enhanced scans. Bilateral neck lymph nodes and larger lymph nodes were observed and showed uniform enhancement on contrast-enhanced scans (**Figures 2F–I**).

**Figure 3:**
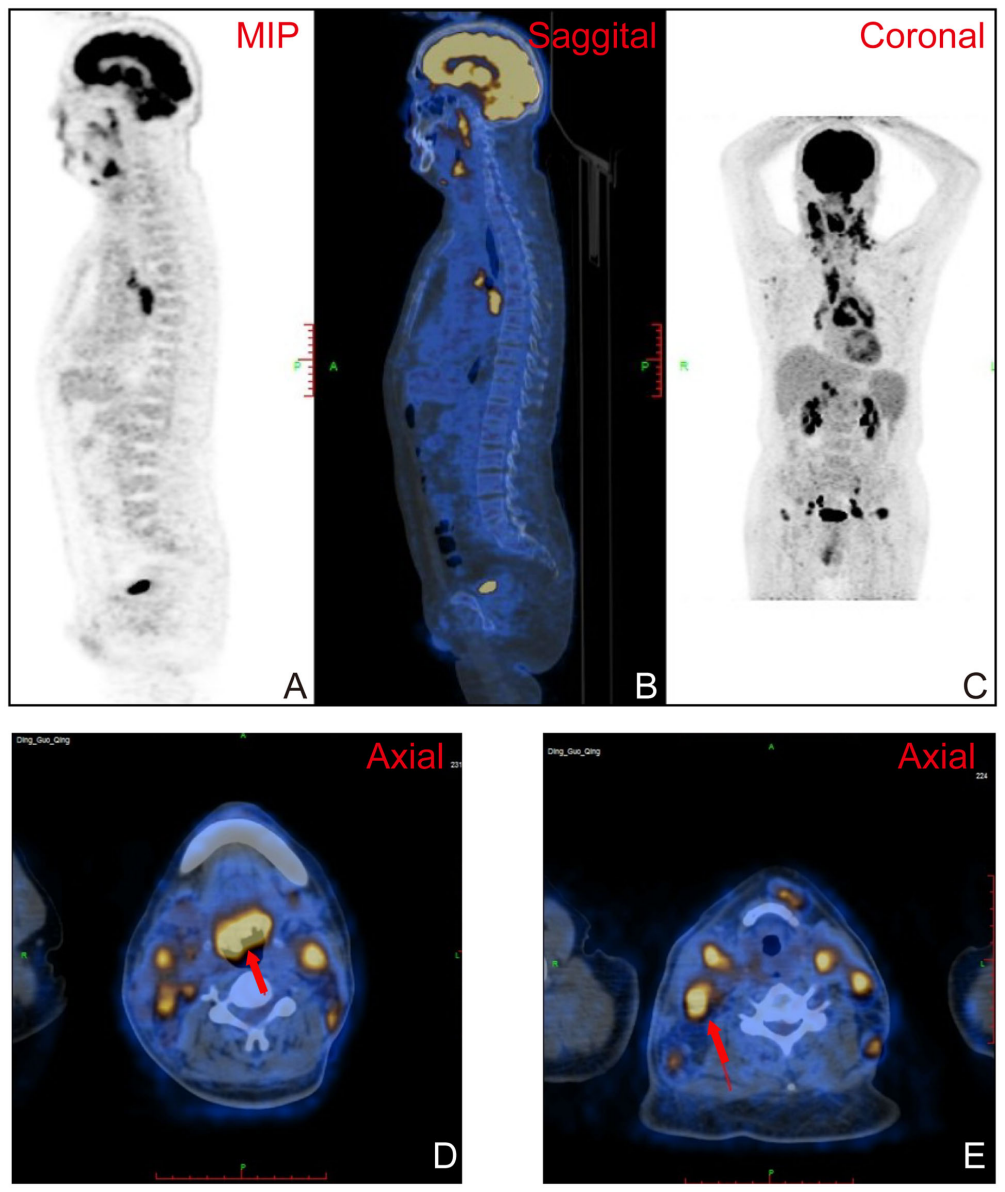
**(A–C)** Positron emission tomography and computed tomography (PET-CT) imaging demonstrating thickening of the soft tissues in the left nasopharyngeal region and parapharyngeal space, accompanied by increased metabolic activity. **(D)** Significant thickening of the soft tissues in tongue base, resulting in narrowing of the pharyngeal cavity (red arrowhead). **(E)** Enlarged lymph nodes in various regions, exhibiting fusion and increased metabolic activity (red arrowhead).

Positron emission tomography and computed tomography (PET-CT) imaging showed that the left nasopharyngeal region and parapharyngeal space demonstrated thickening of the soft tissues, accompanied by increased metabolic activity (**Figures 3A–C**). Significant thickening of the soft tissues was also observed in the right anterior wall of the oropharynx, tongue base, and epiglottis region, leading to the narrowing of the pharyngeal cavity (**Figure 3D**). Enlarged lymph nodes were detected in various regions, showing fusion and increased metabolic activity (**Figure 3E**).

### Pathological examination

**Figure 4:**
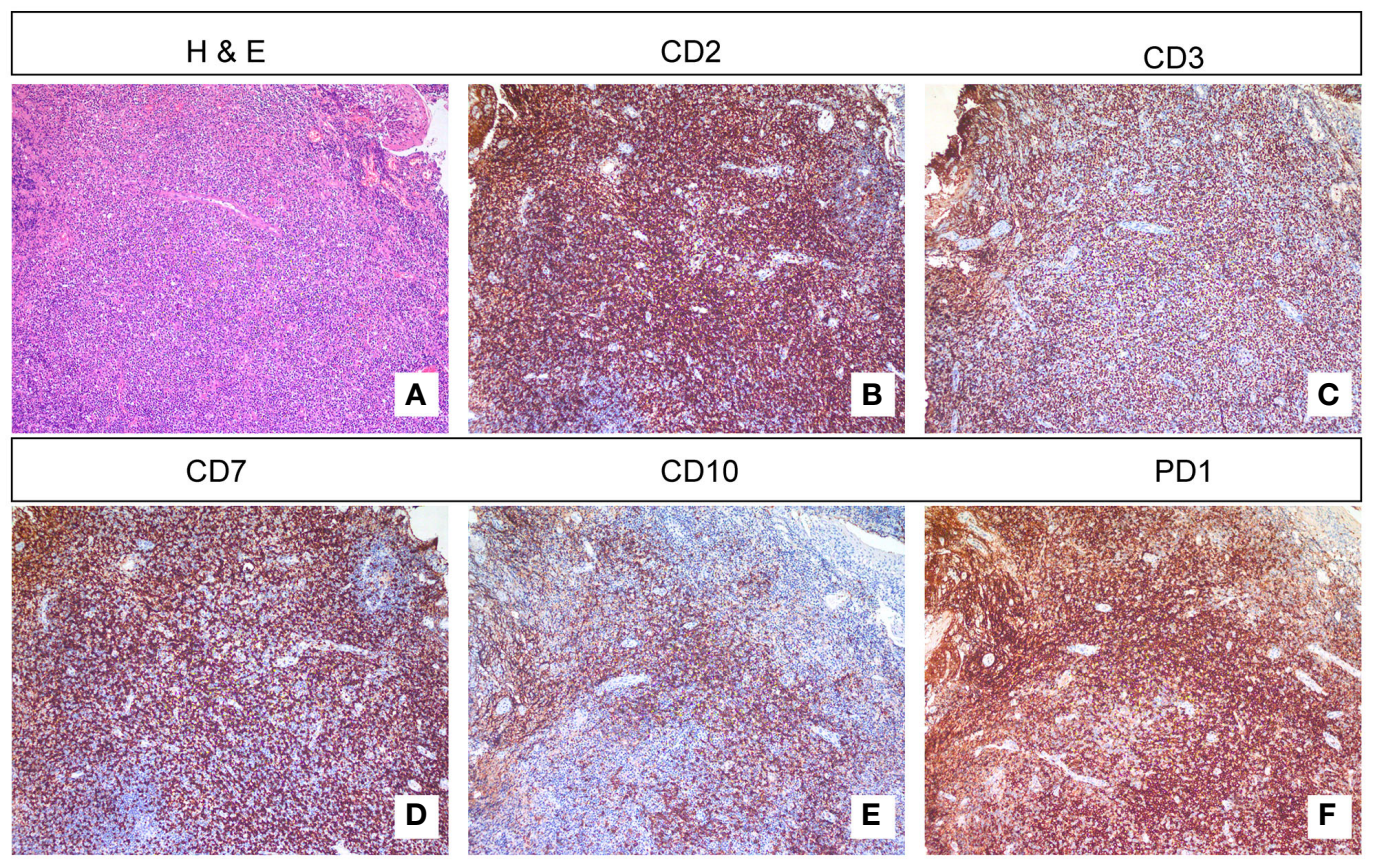
H&E staining and immunohistochemistry. **(A)** H&E staining reveals abundant lymphocytes under the squamous epithelium. **(B, C)** Immunohistochemical staining showed positivity for CD2 and CD3. **(D)** Some T lymphocytes are negative for CD7. **(E)** CD10-positive cells can be found outside the germinal center region. **(F)** Immunohistochemical staining showed positivity for PD1.

The patient underwent a total of three biopsies, with the first two performed at Guizhou Provincial Staff Hospital. Pathological examination from both biopsies revealed chronic inflammation of the epithelial mucosa (data not shown). The third biopsy was conducted at our hospital. The pathology results from the biopsy of the tongue base tissue indicated peripheral T-cell lymphoma with a follicular T-helper cell phenotype. This case represents an extranodal involvement, posing challenges in morphological classification, and is suggestive of angioimmunoblastic T-cell lymphoma. Immunohistochemical staining showed positivity for CD2, CD3, CD5, CD4, PD1, ICOS, CXCL13, CD10, BCL6, CD43, CD21 (FDC+), CD30 (scattered+), and TdT (**Figure 4**) (data shown partially), while it showed negativity for CD7, CD8, MUM1, BCL2, LEF1, cyclin D1, IgD, CD56, and Ki67 (approximately 50% labeling index) (data not shown). *In situ* hybridization testing for Epstein–Barr virus-encoded RNA (EBER) demonstrated positivity (up to 20/HPF), and T-cell receptor gamma (TCRG) clonality was confirmed by the detection of rearranged bands (R23-00052) in the target region.

## Discussion

Lymphoma is the third most common malignancy worldwide, representing 3% of all malignant tumors (6). Lymphomas account for approximately 12% of all malignant tumors in the head and neck region, making them the third most common malignancy after squamous cell carcinoma (46%) and thyroid carcinoma (33%) (7). Laryngeal lymphoma is a rare malignancy, and its clinical presentation often lacks specificity, leading to diagnostic challenges (6). Thus, lymphoma should always be taken into account in cases of unknown cervical or oral masses. This case underscores the importance of considering lymphoma as a potential cause in patients presenting with unknown cervical or oral masses, particularly when symptoms persist or worsen over time.

Imaging techniques, such as CT and MRI, play a critical role in establishing the diagnosis and assessing the extent of laryngeal lymphoma (8). Understanding the characteristic imaging findings can aid in distinguishing it from other laryngeal pathologies, such as squamous cell carcinoma or benign lesions (9).

In our case, CT imaging revealed the presence of an irregular and poorly defined soft tissue mass in the oropharyngeal region, indicating the existence of an abnormal growth. Furthermore, noticeable swelling of the epiglottis was observed, potentially impacting the patient’s airway and respiratory function. Subsequent MRI scans confirmed the presence of a sizable mass in the base of the tongue to the epiglottis region, with extension into the bilateral aryepiglottic folds. The contrast-enhanced scans revealed significant enhancement, suggesting increased vascularity and tumor activity. Furthermore, enlarged lymph nodes exhibiting uniform enhancement indicated possible lymphatic involvement and disease spread.

PET-CT imaging provided additional valuable insights into the disease. It demonstrated thickening of the soft tissues in the left nasopharyngeal region and parapharyngeal space, accompanied by increased metabolic activity, indicative of an active pathological process. Additionally, significant thickening of the soft tissues was observed in the right anterior wall of the oropharynx, tongue base, and epiglottis region, resulting in the narrowing of the pharyngeal cavity. The presence of enlarged lymph nodes with fusion and increased metabolic activity further supported the potential dissemination of the disease to other regions. In summary, the distinctive imaging features observed in laryngeal lymphoma, as visualized through CT, MRI, and PET-CT imaging, aid in accurate diagnosis, assessment of the extent of the disease, and differentiation from other laryngeal pathologies. These imaging modalities provide valuable information for planning appropriate diagnosis and treatment strategies.

The pathological analysis of the biopsy from the base of the tongue, in this case, confirmed the diagnosis of peripheral T-cell lymphoma with a follicular helper T-cell phenotype. The immunohistochemical staining showed positive expression of CD2, CD3, CD5, CD4, PD1, ICOS, CXCL13, CD10, BCL6, and CD43, while it showed negative expression of CD7, CD8, MUM1, BCL2, LEF1, Cyclin D1, IgD, CD56, CD20, and TdT. The presence of EBV *in situ* hybridization positivity and the detection of TCRG clonal rearrangement further supported the diagnosis. These pathological findings provide valuable insights into the molecular and immunophenotypic characteristics of laryngeal lymphoma.

It is important to consider the impact of the hospital’s level of expertise and resources on the diagnosis of laryngeal lymphoma. The initial pathology results from the local hospital indicated chronic inflammation of the mucosa, suggesting a misdiagnosis. However, the subsequent biopsy performed at a tertiary care center, with specialized expertise in diagnosing lymphomas, provided an accurate diagnosis. This highlights the importance of referring complex cases to specialized centers with experienced pathologists, as they can provide more accurate diagnoses and optimize patient management.

In conclusion, laryngeal lymphoma presents with nonspecific clinical manifestations, making its diagnosis challenging. However, advanced imaging techniques, such as PET-CT, play a crucial role in evaluating the extent of the disease. Pathological analysis, including immunohistochemistry and molecular studies, further confirms the diagnosis and provides insights into the specific subtype and molecular characteristics of the lymphoma. Collaborative efforts between primary and specialized centers are essential for accurate diagnosis and appropriate management of laryngeal lymphoma, ensuring optimal patient outcomes.

## Data availability statement

The original contributions presented in the study are included in the article/supplementary material. Further inquiries can be directed to the corresponding authors.

## Ethics statement

The Ethics Committee of Tongji Medical College, Huazhong University of Science and Technology approved this study (IORG No: IORG0003571). The studies were conducted in accordance with the local legislation and institutional requirements. Written informed consent for participation in this study was provided by the participants’ legal guardians/next of kin. Written informed consent was obtained from the individual(s) for the publication of any potentially identifiable images or data included in this article.

## Author contributions

BH: Writing – original draft, Writing – review & editing. JH: Conceptualization, Writing – original draft. RD: Formal analysis, Writing – review & editing. YQ: Methodology, Writing – original draft. HS: Funding acquisition, Resources, Writing – original draft. YZ: Visualization, Writing – original draft.
